# Supplementary material for: Selective sorting and destruction of mitochondrial membrane proteins in aged yeast
Source: eLife. 2016 Apr 20;5:e13943. doi: 10.7554/eLife.13943 (PMC4889329; doi:10.7554/eLife.13943)
Supplement: Supplementary file 2. — DOI: http://dx.doi.org/10.7554/eLife.13943.020 [file elife-13943-supp2.docx]

**Supplementary File 2. Yeast Strains Used in this Study**

| **Strain** | **Genotype** |
| --- | --- |
| UCC11595 | MATa/MATα his3Δ1/his3Δ1 leu2Δ0/leu2Δ0 ura3Δ0/ura3Δ0 lys2Δ0/+ trp1Δ63/+ hoΔ::P_SCW11_-cre-EBD78-NatMX/hoΔ::P_SCW11_-cre-EBD78-NatMX loxP-CDC20-Intron-loxP-HphMX/loxP-CDC20-Intron-loxP-HphMX loxP-UBC9-LOXP-LEU2/loxP-UBC9-LOXP-LEU2 TOM70-eGFP-KanMX/+ VPH1-mCherry-KanMX/+ |
| UCC11621 | MATa/MATα his3Δ1/his3Δ1 leu2Δ0/leu2Δ0 ura3Δ0/ura3Δ0 lys2Δ0/+ trp1Δ63/+ hoΔ::P_SCW11_-cre-EBD78-NatMX/hoΔ::P_SCW11_-cre-EBD78-NatMX loxP-CDC20-Intron-loxP-HphMX/loxP-CDC20-Intron-loxP-HphMX loxP-UBC9-LOXP-LEU2/loxP-UBC9-LOXP-LEU2 TOM70-eGFP-KanMX/+ VPH1-mCherry-KanMX/+ atg5Δ::URA3/atg5Δ::URA3 |
| UCC11613 | MATa/MATα his3Δ1/his3Δ1 leu2Δ0/leu2Δ0 ura3Δ0/ura3Δ0 lys2Δ0/+ trp1Δ63/+ hoΔ::P_SCW11_-cre-EBD78-NatMX/hoΔ::P_SCW11_-cre-EBD78-NatMX loxP-CDC20-Intron-loxP-HphMX/loxP-CDC20-Intron-loxP-HphMX loxP-UBC9-LOXP-LEU2/loxP-UBC9-LOXP-LEU2 TOM70-eGFP-KanMX/+ VPH1-mCherry-KanMX/+ dnm1Δ::URA3/dnm1Δ::URA3 |
| UCC11615 | MATa/MATα his3Δ1/his3Δ1 leu2Δ0/leu2Δ0 ura3Δ0/ura3Δ0 lys2Δ0/+ trp1Δ63/+ hoΔ::P_SCW11_-cre-EBD78-NatMX/hoΔ::P_SCW11_-cre-EBD78-NatMX loxP-CDC20-Intron-loxP-HphMX/loxP-CDC20-Intron-loxP-HphMX loxP-UBC9-LOXP-LEU2/loxP-UBC9-LOXP-LEU2 TOM70-eGFP-KanMX/+ VPH1-mCherry-KanMX/+ atg32Δ::URA3/atg32Δ::URA3 |
| UCC11617 | MATa/MATα his3Δ1/his3Δ1 leu2Δ0/leu2Δ0 ura3Δ0/ura3Δ0 lys2Δ0/+ trp1Δ63/+ hoΔ::P_SCW11_-cre-EBD78-NatMX/hoΔ::P_SCW11_-cre-EBD78-NatMX loxP-CDC20-Intron-loxP-HphMX/loxP-CDC20-Intron-loxP-HphMX loxP-UBC9-LOXP-LEU2/loxP-UBC9-LOXP-LEU2 TOM70-eGFP-KanMX/+ VPH1-mCherry-KanMX/+ vam3Δ::URA3/vam3Δ::URA3 |
| UCC11619 | MATa/MATα his3Δ1/his3Δ1 leu2Δ0/leu2Δ0 ura3Δ0/ura3Δ0 lys2Δ0/+ trp1Δ63/+ hoΔ::P_SCW11_-cre-EBD78-NatMX/hoΔ::P_SCW11_-cre-EBD78-NatMX loxP-CDC20-Intron-loxP-HphMX/loxP-CDC20-Intron-loxP-HphMX loxP-UBC9-LOXP-LEU2/loxP-UBC9-LOXP-LEU2 TOM70-eGFP-KanMX/+ VPH1-mCherry-KanMX/+ pep4Δ::URA3/pep4Δ::URA3 |
| UCC11843 | MATa/MATα his3Δ1/his3Δ1 leu2Δ0/leu2Δ0 ura3Δ0/ura3Δ0 lys2Δ0/+ trp1Δ63/+ hoΔ::P_SCW11_-cre-EBD78-NatMX/hoΔ::P_SCW11_-cre-EBD78-NatMX loxP-CDC20-Intron-loxP-HphMX/loxP-CDC20-Intron-loxP-HphMX loxP-UBC9-LOXP-LEU2/loxP-UBC9-LOXP-LEU2 TOM70-eGFP-KanMX/+ VPH1-mCherry-KanMX/+ pep4Δ::SpHIS5MX/pep4Δ::SpHIS5MX |
| UCC11849 | MATa/MATα his3Δ1/his3Δ1 leu2Δ0/leu2Δ0 ura3Δ0/ura3Δ0 lys2Δ0/+ trp1Δ63/+ hoΔ::P_SCW11_-cre-EBD78-NatMX/hoΔ::P_SCW11_-cre-EBD78-NatMX loxP-CDC20-Intron-loxP-HphMX/loxP-CDC20-Intron-loxP-HphMX loxP-UBC9-LOXP-LEU2/loxP-UBC9-LOXP-LEU2 TOM70-eGFP-KanMX/+ VPH1-mCherry-KanMX/+ pep4Δ::SpHIS5MX/pep4Δ::SpHIS5MX atg5Δ::URA3/atg5Δ::URA3 |
| UCC11845 | MATa/MATα his3Δ1/his3Δ1 leu2Δ0/leu2Δ0 ura3Δ0/ura3Δ0 lys2Δ0/+ trp1Δ63/+ hoΔ::P_SCW11_-cre-EBD78-NatMX/hoΔ::P_SCW11_-cre-EBD78-NatMX loxP-CDC20-Intron-loxP-HphMX/loxP-CDC20-Intron-loxP-HphMX loxP-UBC9-LOXP-LEU2/loxP-UBC9-LOXP-LEU2 TOM70-eGFP-KanMX/+ VPH1-mCherry-KanMX/+ pep4Δ::SpHIS5MX/pep4Δ::SpHIS5MX dnm1Δ::URA3/dnm1Δ::URA3 |
| UCC11847 | MATa/MATα his3Δ1/his3Δ1 leu2Δ0/leu2Δ0 ura3Δ0/ura3Δ0 lys2Δ0/+ trp1Δ63/+ hoΔ::P_SCW11_-cre-EBD78-NatMX/hoΔ::P_SCW11_-cre-EBD78-NatMX loxP-CDC20-Intron-loxP-HphMX/loxP-CDC20-Intron-loxP-HphMX loxP-UBC9-LOXP-LEU2/loxP-UBC9-LOXP-LEU2 TOM70-eGFP-KanMX/+ VPH1-mCherry-KanMX/+ pep4Δ::SpHIS5MX/pep4Δ::SpHIS5MX atg32Δ::URA3/atg32Δ::URA3 |
| UCC4961 | MATa/MATα his3Δ1/his3Δ1 leu2Δ0/leu2Δ0 ura3Δ0/ura3Δ0 lys2Δ0/+ trp1Δ63/+ hoΔ::P_SCW11_-cre-EBD78-NatMX/hoΔ::P_SCW11_-cre-EBD78-NatMX loxP-CDC20-Intron-loxP-HphMX/loxP-CDC20-Intron-loxP-HphMX loxP-UBC9-LOXP-LEU2/loxP-UBC9-LOXP-LEU2 TOM70-mCherry-KanMX/+ |
| UCC4965 | MATa/MATα his3Δ1/his3Δ1 leu2Δ0/leu2Δ0 ura3Δ0/ura3Δ0 lys2Δ0/+ trp1Δ63/+ hoΔ::P_SCW11_-cre-EBD78-NatMX/hoΔ::P_SCW11_-cre-EBD78-NatMX loxP-CDC20-Intron-loxP-HphMX/loxP-CDC20-Intron-loxP-HphMX loxP-UBC9-LOXP-LEU2/loxP-UBC9-LOXP-LEU2 TOM70-mCherry-KanMX/+ TIM50-eGFP-KanMX/+ |
| UCC4935 | MATa/MATα his3Δ1/his3Δ1 leu2Δ0/leu2Δ0 ura3Δ0/ura3Δ0 lys2Δ0/+ trp1Δ63/+ hoΔ::P_SCW11_-cre-EBD78-NatMX/hoΔ::P_SCW11_-cre-EBD78-NatMX loxP-CDC20-Intron-loxP-HphMX/loxP-CDC20-Intron-loxP-HphMX loxP-UBC9-LOXP-LEU2/loxP-UBC9-LOXP-LEU2 TOM70-mCherry-KanMX/+ DNM1-eGFP-KanMX/+ |
| AHY1033 | MATa/MATα his3Δ1/his3Δ1 leu2Δ0/leu2Δ0 ura3Δ0/ura3Δ0 lys2Δ0/+ trp1Δ63/+ hoΔ::P_SCW11_-cre-EBD78-NatMX/hoΔ::P_SCW11_-cre-EBD78-NatMX loxP-CDC20-Intron-loxP-HphMX/loxP-CDC20-Intron-loxP-HphMX loxP-UBC9-LOXP-LEU2/loxP-UBC9-LOXP-LEU2 TOM70-mCherry-KanMX/+ chrI(199456-199457)::P_GPD1_-eGFP-ATG8-Term_CYC1_-URA3/chrI(199456-199457)::P_GPD1_-eGFP-ATG8-Term_CYC1_-URA3 |
| AHY705 | MATa/MATα his3Δ1/his3Δ1 leu2Δ0/leu2Δ0 ura3Δ0/ura3Δ0 lys2Δ0/+ trp1Δ63/+ hoΔ::P_SCW11_-cre-EBD78-NatMX/hoΔ::P_SCW11_-cre-EBD78-NatMX loxP-CDC20-Intron-loxP-HphMX/loxP-CDC20-Intron-loxP-HphMX loxP-UBC9-LOXP-LEU2/loxP-UBC9-LOXP-LEU2 TOM70-mCherry-KanMX/+ TOM20-eGFP-KanMX/+ |
| UCC11775 | MATa/MATα his3Δ1/his3Δ1 leu2Δ0/leu2Δ0 ura3Δ0/ura3Δ0 lys2Δ0/+ met15Δ0/+ Term_CYC1_:URA3-P_GPD/TDH3_-cre-EBD78:Term_CYC1_/+ TOM70-V5-loxP-HA-GFP-HphMX-loxP-T7-mRFP-KanMX/+ |
| UCC11785 | MATa/MATα his3Δ1/his3Δ1 leu2Δ0/leu2Δ0 ura3Δ0/ura3Δ0 lys2Δ0/+ met15Δ0/+ Term_CYC1_:URA3-P_GPD/TDH3_-cre-EBD78:Term_CYC1_/+ OAC1-V5-loxP-HA-GFP-HphMX-loxP-T7-mRFP-KanMX/+ |
| UCC4997 | MATα his3Δ1 leu2Δ0 ura3Δ0 lys2Δ0 TOM70-mCherry-KanMX |
| BY4741 | MATa his3Δ1 leu2Δ0 ura3Δ0 met15Δ0 |
| AHY1657 | MATa his3Δ1 leu2Δ0 ura3Δ0 met15Δ0 tom70Δ::URA3 |
| AHY1665 | MATa his3Δ1 leu2Δ0 ura3Δ0 met15Δ0 tom70Δ::URA3 tom71Δ::LEU2 |
| AHY1669 | MATa his3Δ1 leu2Δ0 ura3Δ0 met15Δ0 COX7-eGFP-KanMX |
| AHY1671 | MATa his3Δ1 leu2Δ0 ura3Δ0 met15Δ0 tom70Δ::URA3 COX7-eGFP-KanMX |
| AHY1676 | MATa his3Δ1 leu2Δ0 ura3Δ0 met15Δ0 tom70Δ::URA3 tom71Δ::LEU2 COX7-eGFP-KanMX |
| AHY1683 | MATa his3Δ1 leu2Δ0 ura3Δ0 met15Δ0 COX7-eGFP-KanMX dnm1Δ::HpHMX |
| AHY1685 | MATa his3Δ1 leu2Δ0 ura3Δ0 met15Δ0 tom70Δ::URA3 COX7-eGFP-KanMX dnm1Δ::HpHMX |
| AHY1687 | MATa his3Δ1 leu2Δ0 ura3Δ0 met15Δ0 tom70Δ::URA3 tom71Δ::LEU2 COX7-eGFP-KanMX dnm1Δ::HpHMX |
| AHY689 | MATa/MATα his3Δ1/his3Δ1 leu2Δ0/leu2Δ0 ura3Δ0/ura3Δ0 lys2Δ0/+ met15Δ0/+ fis1Δ::KanMX/fis1Δ::KanMX TOM70-eGFP-SpHIS5MX/+ |
