# Supplementary material for: Selective sorting and destruction of mitochondrial membrane proteins in aged yeast
Source: eLife. 2016 Apr 20;5:e13943. doi: 10.7554/eLife.13943 (PMC4889329; doi:10.7554/eLife.13943)
Supplement: Supplementary file 3. — DOI: http://dx.doi.org/10.7554/eLife.13943.021 [file elife-13943-supp3.docx]

**Supplementary File 3.** **Oligos Used in this Study**

| **Name** | **Sequence** |
| --- | --- |
| Tom70 F5 | TCAAGAAACTTTAGCTAAATTACGCGAACAGGGTTTAATGGGTGACGGTGCTGGTTTA |
| Tom70 R3 | TTTGTCTTCTCCTAAAAGTTTTTAAGTTTATGTTTACTGTTCGATGAATTCGAGCTCG |
| Tom70 F5 Cherry | TCAAGAAACTTTAGCTAAATTACGCGAACAGGGTTTAATGggtcgacggatccccggg |
| Vph1 F5 Cherry | GGAAGTCGCTGTTGCTAGTGCAAGCTCTTCCGCTTCAAGCggtcgacggatccccggg |
| Vph1 R3 | AGTACTTAAATGTTTCGCTTTTTTTAAAAGTCCTCAAAATTCGATGAATTCGAGCTCG |
| Tim50 F5 | TGAAGAGGAAAAGAAAAAGAAGAAGATTGCTGAATCCAAAGGTGACGGTGCTGGTTTA |
| Tim50 R3 | ATAGATACGTAGATACATGAGAAGAGGGTTTACATGAAAATCGATGAATTCGAGCTCG |
| Tom20 F5 | GCCGAATCTGATGCGGTTGCTGAAGCTAACGATATCGATGACGGTGACGGTGCTGGTTTA |
| Tom20 R3 | AAGAAACAAAAACGGAGAAAAAAAGCAAGCAAAATGTTACTCTCGATGAATTCGAGCTCG |
| Dnm1 F5 | AGTTTATAAAAAGGCTGCAACCCTTATTAGTAATATTCTGGGTGACGGTGCTGGTTTA |
| Dnm1 R3 | CAATGTTGAAGTAAGATCAAAAATGAGATGAATTATGCAATCGATGAATTCGAGCTCG |
| Cox7 F5 | CATTCCAAATGCTATTAGAGGTATCAAAGCCAAGAAGGCAGGTGACGGTGCTGGTTTA |
| Cox7 R3 | AATTATAAAGATGCTATGAACGGATGTTATTTTTACCTAATCGATGAATTCGAGCTCG |
| Tom70 F5 RITE | ATTCAAGAAACTTTAGCTAAATTACGCGAACAGGGTTTAATGGGTGGATCTGGTGGATCT |
| Tom70 R3 RITE | TTTTTGTCTTCTCCTAAAAGTTTTTAAGTTTATGTTTACTGTGGCGCCGGTGGAGTGGCG |
| Oac1 F5 RITE | AAACTAGTTTATTCGATAGAGTCGAGAGTTTTAGGCCATAATGGTGGATCTGGTGGATCT |
| Oac1 R3 RITE | GCCAATGAATGAAACTTCAAACCTCGGAGTTTGTTATGGGAAGGCGCCGGTGGAGTGGCG |
| Tom70 D5 | GAAGTGAAATTACAGCTCACATCTAGGTTCTCAATTGCCAGATTGTACTGAGAGTGCACC |
| Tom70 D3 | TTTGTCTTCTCCTAAAAGTTTTTAAGTTTATGTTTACTGTCTGTGCGGTATTTCACACCG |
| Tom71 D5 | ATCTCTACATACTTGTATATACCGAACATAAGAAGCTCTTAGATTGTACTGAGAGTGCAC |
| Tom71 D3 | TAACTAAAAGTATATATTTGACCAATACCTGACATATCTTCTGTGCGGTATTTCACACCG |
| Atg5 D5 | GGTTCTAGAAGAACGGAGATAGGAAACCTATGATGTAAGTGATTGTACTGAGAGTGCACC |
| Atg5 D3 | GATATTTGAATGACACTTTTAAATGCGTATATAACAGCTCCTGTGCGGTATTTCACACCG |
| Dnm1 D5 | TTAAGTAGCTACCAGCGAATCTAAATACGACGGATAAAGAGATTGTACTGAGAGTGCACC |
| Dnm1 D3 | TGTTGAAGTAAGATCAAAAATGAGATGAATTATGCAATTACTGTGCGGTATTTCACACCG |
| Atg32 D5 | ATCACAAAAGCAAAAAAAATCTGCCAGGAACAGTAAACATGATTGTACTGAGAGTGCACC |
| Atg32 D3 | GTGAGTAGGAACGTGTATGTTTGTGTATATTGGAAAAAGGCTGTGCGGTATTTCACACCG |
| Pep4 D5 | ATTTAATCCAAATAAAATTCAAACAAAAACCAAAACTAACGATTGTACTGAGAGTGCACC |
| Pep4 D3 | GGCAGAAAAGGATAGGGCGGAGAAGTAAGAAAAGTTTAGCCTGTGCGGTATTTCACACCG |
| Vam3 D5 | CAAATTGGCCAACTAATATCCACTGCAGAAAGTTGAGATTGATTGTACTGAGAGTGCACC |
| Vam3 D3 | TACCAGAAAGTCTGTGCTCAATGCGCGTTTAAGGAGATTACTGTGCGGTATTTCACACCG |
| Pep4 D5 His5MX | ATTTAATCCAAATAAAATTCAAACAAAAACCAAAACTAACGACAGTCTTGACGTGCGC |
| Pep4 D3 His5MX | GGCAGAAAAGGATAGGGCGGAGAAGTAAGAAAAGTTTAGCTCGATGAATTCGAGCTCG |
| ChrI PartA NotI F | GTCCCATTCGAAGAAGCGGCCGCTTTAGCTCATTGAGATATGTG |
| ChrI PartA SmaI R | GTATTCTACGACCCCGGGGGTGCTAATTATGGCATTGAT |
| ChrI PartB SmaI F | CGTCAATGCAAGCCCGGGCCATGGATGGTCGTTTAAGGC |
| ChrI PartB NotI R | TCTCAATGAGCTAAAGCGGCCGCTTCTTCGAATGGGACCAGCTA |
